# Effect of Tin Grain Orientation on Electromigration-Induced Dissolution of Ni Metallization in SnAg Solder Joints

**DOI:** 10.3390/ma15207115

**Published:** 2022-10-13

**Authors:** Po-Ning Hsu, Dai-Lung Lee, Dinh-Phuc Tran, Kai-Cheng Shie, Nien-Ti Tsou, Chih Chen

**Affiliations:** 1 Department of Materials Science and Engineering, National Yang Ming Chiao Tung University, Hsinchu 30010, Taiwan; 2 Department of Materials Science and Engineering, National Chiao Tung University, Hsinchu 30010, Taiwan

**Keywords:** electromigration, grain orientation, UBM consumption, diffusion

## Abstract

In this study, symmetrical solder joints (Cu/Ni/SnAg_2.3_/Ni/Cu) were fabricated. They were electromigration (EM)-stressed at high (8 × 10^4^ A/cm^2^) or low (1.6 × 10^4^ A/cm^2^) current densities. Failures in the solder joints with different grain orientations under EM stressing were then characterized. Results show that Ni under-bump-metallurgy (UBM) was quickly dissolved into the solder joints possessing low angles between Sn *c*-axis and electron direction and massive NiCuSn intermetallic compounds formed in the Sn matrix. The diffusion rate of Ni increased with decreasing orientation grain angle. A theoretical model was also established to analyze the consumption rate of Ni UBM. Good agreement between the modeling and experimental results was obtained. Additionally, we found that voids were more likely to form in the solder joints under high EM stressing.

## 1. Introduction

Electromigration (EM) is a critical reliability issue in solder joints [1,2,3,4]. The nickel (Ni) metal in cathode will dissolve into solder to form intermetallic compounds (IMCs) such as (Ni,Cu)_3_Sn_4_ and/or (Cu,Ni)_6_Sn_5_. In the microelectronic packaging, nickel-based metallization schemes have been widely used, and the most common fabrication technique used is transient liquid phase (TLP) bonding. Considering some aspects such as bonding strength and fabrication cost, IMCs generated during low-temperature bonding are widely favored because of the excellent bonding strength of solder joints [5,6,7,8]. It has been reported that the diffusion rate of Ni into solder at high temperatures in *c*-axis direction is four orders greater than that in *a*-axis or *b*-axis [9]. Therefore, the grain orientation of solder joints could greatly affect IMC formation under EM stressing. Currently, the effect of grain orientation on IMC formation in solder joints during thermomigration (TM) has been intensively investigated [10,11,12,13]. However, studies on the correlation between grain orientation and EM-induced failures are limited.

During fabrication processes, solders are commonly heated up to their melting point [14,15]. Ni under-bump metallization (UBM) and Cu react with solder during reflow soldering [16,17,18]. According to Ni-Sn and Cu-Sn phase diagrams, various IMCs such as Ni_3_Sn_4_, Ni_3_Sn_2_, Ni_3_Sn, Cu_6_Sn_5_ and Cu_3_Sn form [19,20,21]. Silver (Ag) is widely used in solder to inhibit the formation of microvoids [22] and the growth of brittle IMCs [23,24]. It is also adopted to enhance the mechanical strength [23,25,26] and resistance to thermal fatigue of solders [27,28,29]. However, Yang et al. reported that an excessive amount of Ag added into solder (>3.5 wt.%) may lead to high stress concentration [18,30,31]. Plate-like Ag-Sn IMCs will form, and cracks may initiate along the interface resulting in solder failures [30,32]. Thus, in the electronic packaging technology, an optimal concentration of Ag (2.4–3.5 wt.%) is recommended to reduce the formation of such IMCs [33]. Previously, Wang et al. showed that the shear strength of solder joints increased as IMCs grew to a certain thickness [34]. Once reaching a certain turning point, various voids appeared leading to the decrease of its strength. Coincidentally, grain refinement could happen and cause void formation when the density of Ni-containing IMCs reaches a certain level [35]. Recently, Qiao et al. showed that the concentration of Cu atoms within IMCs could affect the inconsistency of the growth rates of hot and cold microbump ends. This could be attributed to the strong anisotropy diffusion of Cu atoms in *β*-Sn grains [36]. The crystal structure of β-Sn is body-centered tetragonal (BCT), and its lattice constants are *a* = *b* = 5.83 Å and *c* = 3.18 Å.

In this study, symmetrical solder joints (Cu/Ni/SnAg_2.3_/Ni/Cu) were fabricated. The joints were stressed at high (8 × 10^4^ A/cm^2^) and low (1.6 × 10^4^ A/cm^2^) current densities. The failures mechanisms of solder joints with different Sn grain orientations under EM stressing were characterized. A theoretical model is established to analyze the dissolution rate of Ni and correlate with the grain orientation of the solder joints.

## 2. Materials and Methods

**Figure 1:**
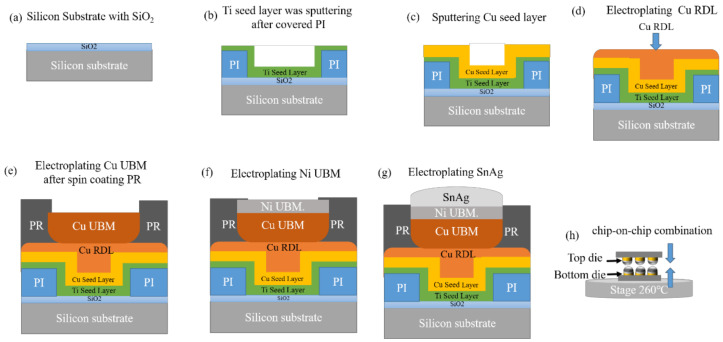
Flow chart of the sample fabrication: (**a**) 8-inch wafer as substrate; (**b**) sputtering PI and Ti seed layer; (**c**) sputtering Cu seed layers; (**d**) electroplating the Cu RDL; (**e**) electroplating Cu UBM after spin-coating PR; (**f**) electroplating a Ni UBM layer; (**g**) electroplating SnAg; (**h**) flip-chip solder joints were bonded by thermal compression.

**Figure 2:**
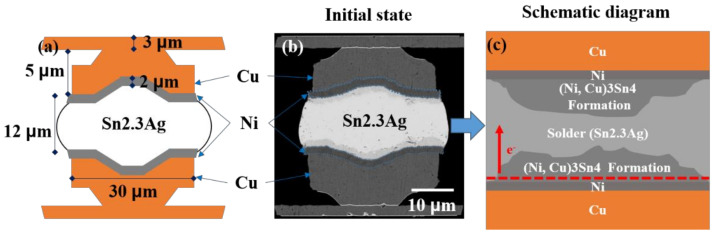
Dimension and structure of the solder microbumps used in this study: (**a**) schematic drawing of the SnAg microbumps; (**b**) as-fabricated SnAg microbump with Ni/Cu UBM. (**c**) Schematic diagram of the microbumps showing the induced phases and atomic diffusion of Ni flow under EM stressing.

Various symmetrical solder joints (Cu/Ni/SnAg_2.3_/Ni/Cu) were fabricated on a silicon substrate. Detailed fabrication processes are shown in Figure 1. An 8-inch wafer was used as the substrate for depositing Ti and Cu seed layers with thicknesses of 0.1 and 0.3 μm, respectively. A Cu redistribution line (RDL) was then electrodeposited after spin coating polyimide (PI). Next, Cu and Ni UBM layers were electroplated after spin-coating photoresist (PR). Subsequently, SnAg solders were electroplated on the Ni UBMs. Finally, the chips were placed face to face and aligned. Then flip-chip solder joints were fabricated using the thermal compression bonding (TCB) process. The melting point of the SnAg solder is approximately 230 °C. The bonding condition was under a pressure of 10 N with a bonding time of 3 s at 250 °C. Then, the samples were cooled with air under a cooling rate of approximately 10 °C/s. In this study, each solder joint consisted of 5 μm thick Cu, 2 μm thick Ni and 12 μm thick SnAg_2.3_. The diameter and pitch of the joints were 30 and 80 μm, respectively. During the bonding processes, the molten solder tended to reduce its surface tension. Thus, it formed a semi-sphere shape on Ni UBM. Figure 2a shows the dimensions of the solder microbump adopted in this study. The fabricated specimen is presented in Figure 2b. An schematic diagram of the sample structure is shown in Figure 2c.

**Figure 3:**
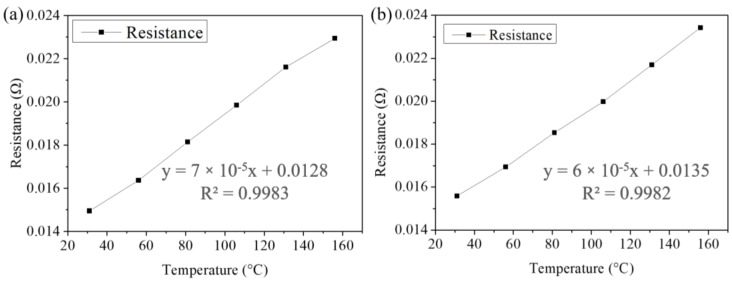
Relationship between hotplate temperature and resistance change during the TCR tests of (**a**,**b**) two solder joints using a Kelvin structure.

The microbumps were stressed at a high-current density of 8 × 10^4^ A/cm^2^ or low-current density of 1.6 × 10^4^ A/cm^2^ on a heating plate (150 °C). During EM testing, the solder joints were heated to 150 °C on a hotplate, and the electrical resistance of the joints was monitored. However, Joule heating was induced, leading to an increase in sample temperature. The actual temperature in the solder joints subjected was higher than the set value (150 °C). Therefore, we employed a thermocouple to calibrate the temperature coefficient of resistance (TCR) and to correct the actual temperature in the solder joints. This method was used to calculate the relative change of electrical resistance for each degree of temperature increase. An electrical current of 0.02 A was used. A temperature interval of 25 °C was set until reaching 156 °C. First, we divided the temperature into 6 parts, measured each 26 °C for 600 s, and took an average of all the resistance values. Then, six points of the resistances of the samples were monitored, and linear regression was applied. Then, the resistance value could be obtained, as shown in Figure 3.

After a certain resistance change, the test samples were then cross-sectioned and polished for scanning electron microscopy (SEM, 6500-SEM, JEOL, Japan) and backscattered electron diffraction (EBSD, Oxford Instruments, Abingdon, UK) analyses. With these analyses, the α-angles between electron direction and the c-axis of solder grains were correlated with EM-induced failures.

## 3. Results and Discussion

**Figure 4:**
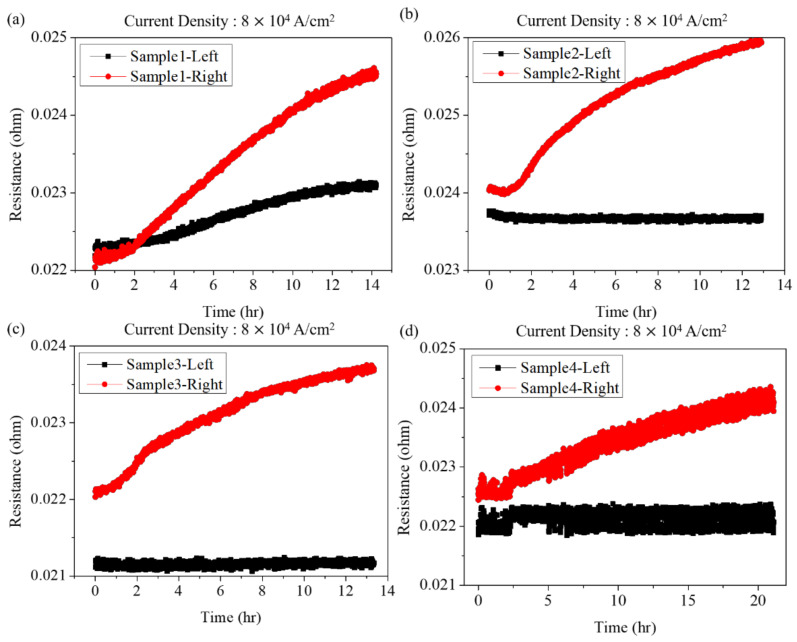
The resistance changes of (**a**) sample-1 (**b**) sample-2 (**c**) sample-3, and (**d**) sample-4 under a high current density.

**Figure 5:**
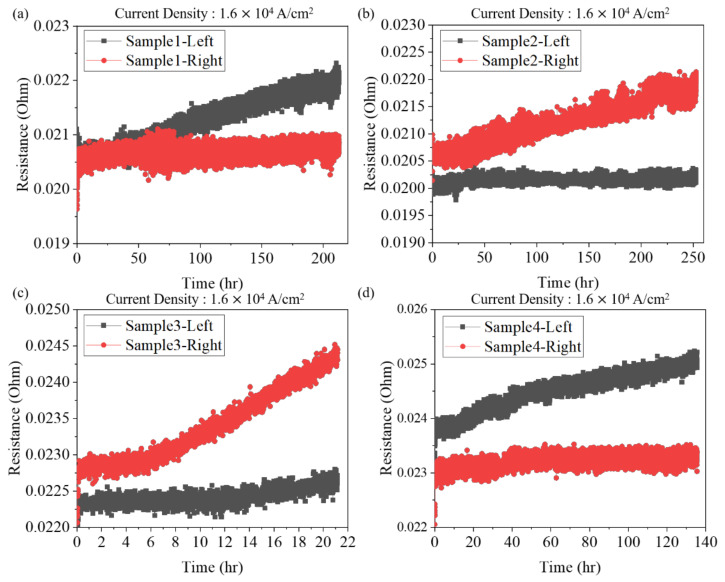
The resistance changes of (**a**) sample-1 (**b**) sample-2 (**c**) sample-3, and (**d**) sample-4 under a low current density.

Under EM stressing, the metallurgical Ni layer at cathode end quickly dissolved into the solder to form the IMCs ((Ni,Cu)_3_Sn_4_ and/or (Cu,Ni)_6_Sn_5_). Figure 4 shows the resistance change of the solder joints under a high current density. The Ni layer was the source for the formation of CuNiSn IMCs. We could calculate the theoretical dissolution rate of the Ni UBM to analyze the damage of the joints under EM stressing. The change of electrical resistance of the joints under a low current density is shown in Figure 5. The resistance increased with the increase of stressing time. The resistance increase may be attributed to the formation of IMCs and voids.

**Figure 6:**
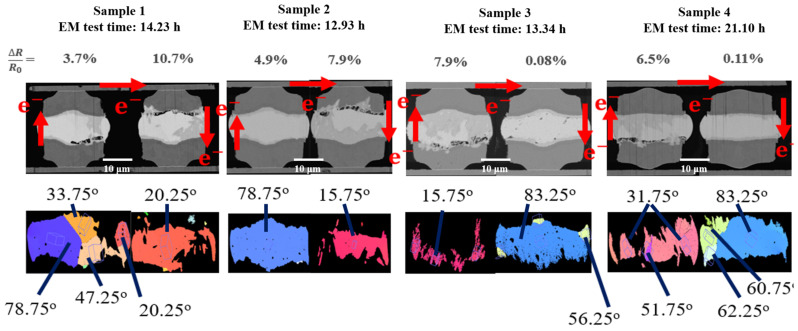
Cross-sectional FIB and EBSD images of the solder joints with different α-angle stressed at a high current density. The electrical flows are denoted by the red arrows.

**Figure 7:**
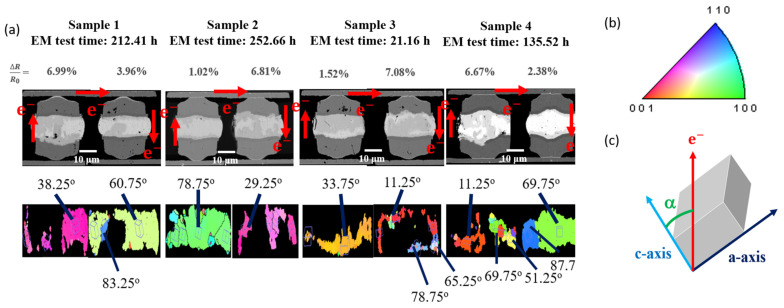
(**a**) Cross-sectional FIB and EBSD images of the solder joints with different α-angle stressed at a low current density. (**b**) Inverse pole figure of the β-Sn. (**c**) Schematic diagram showing the definition of α-angle. The electrical flows are denoted by the red arrows.

First, we define the direction of electron flow, as shown in the upper images in Figure 6 and Figure 7. The electron flow versus angle α and solder grain c-axis were used to correlate the grain orientation and EM failures, as shown in Figure 7c. The angles of the orientation of the tin grains were then scanned using EBSD. The inverse pole diagram of β-Sn is shown in Figure 7b. The results of the samples with high current density are shown in Figure 6. From the FBI images, it can be observed that the electron flow is from bottom to top to the solder microbumps on the right, and the electron flow is from top to bottom, which is damaged by EM. The area is the lower right corner of the left bump and just above the right bump. We can observe that the electron flow enters from the lower angle area by comparing the angles. Although the microbumps on the left side of sample 2 did not produce EM damage, the overall grain orientation angle was relatively high, which was not observed here. Samples 1, 3 and 4 all have the same performance as the micro-bumps on the left side. At lower angles, the grains were prone to EM-induced damage by the electron flow. 

The test results of samples with low current density applied are shown in Figure 7a. From the FBI picture, it can be observed that the electron flow was from bottom to top to the solder micro-bump on the right, and the electron flow was from top to bottom. The damaged area of sample 1 affected by EM is the left micro-bump below the center, and sample 4 was in the lower right corner. The remaining samples 2 and 3 were not damaged by EM. Analyzing the solder that has not been converted into an intermetallic compound after electromigration, we can observe that the microstructure of the joint was examined after its resistance reached a certain value. In this study, the resistance increase was less than 10%, so an initial failure mechanism could be observed. Numerous voids were found at the cathode of the joints. At a high α-angle, that is, when the angle between the electron flow direction and the *c*-axis of the solder grain was high, it was more resistant to damage caused by electromigration electron wind, and the remaining metal nickel pad was thicker. However, at a low α-angle, it was less resistant to the damage caused by the electron wind, and the remaining metal nickel cushion layer was thinner.

The orientation of Sn grains plays an important role in the diffusion of metal UBMs [37]. They anisotropically diffuse in solder resulting in two different failures. First, when the electron direction is parallel to the *c*-axis of Sn grains, the atoms in metal UBM rapidly dissolve and react with solder to form IMCs. The excessive consumption of metal UBM produces more IMCs. In the later stage, Cu wire is consumed and replaced by solder. Second, when the electron flow is perpendicular to the solder *c*-axis, the UBM atoms are hard to diffuse into the solder. As Sn atoms are pushed away by EM wind force, no proper amount of UBM atoms is to fill the produced gaps, resulting in the formation of voids [38].

**Figure 8:**
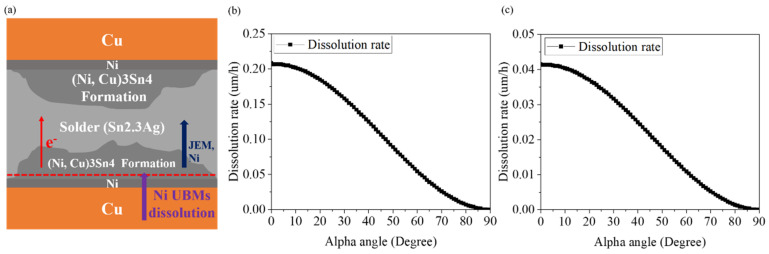
(**a**) Schematic diagram showing the atomic diffusion of Ni flow. The red dotted line represents the original position before the EM test. Relationship between the theoretical dissolution rate of Ni UBM and *α*-angle at (**b**) 8 × 10^4^ A/cm^2^ and (**c**) 1.6 × 10^4^ A/cm^2^ current densities.

As shown in Figure 8a, the original thickness of Ni UBM on the cathode end is labeled by the red dotted line. The thickness of the Ni layer was reduced due to EM-driven dissolution. Thus, the number of Ni atoms diffusing to the solder anode can be estimated by the thickness variation of the Ni UBM. Since the source of Ni in the CuNiSn IMCs is mainly from the Ni UBM on the cathode end, we can assume the number of atoms diffused from the Ni UBM as,

(1) $$\mathrm{Ni}\;\mathrm{atoms}=\mathrm{atomic}\;\mathrm{flux}\;(\mathrm{atoms}/{\mathrm{cm}}^{2}/s)\times \mathrm{area}\;({\mathrm{cm}}^{2})\times \mathrm{time}\;(s)$$

The atomic flow of Ni per time unit in solder with an *α*-angle can be expressed as,

(2) $$J_{\mathrm{EM},\mathrm{Ni}}=C_{\mathrm{Ni}}\times \frac{D_{\mathrm{Ni}(\alpha )}}{kT}\times \left(Z^{*}\times e\times \rho_{\mathrm{Sn}}\times j\right)$$

where *C*_Ni_ is the concentration of Ni in solder (1/cm^3^), *D*_Ni(α)_ is the diffusion coefficient of Ni in solder with an *α*-angle, *k* is the Boltzman constant (J/K), *T* is the experimental temperature (K), Z* is the number of effective charges, *e* is the electron charge (C), *ρ*_Sn_ is the resistivity of Sn (Ω-cm), and *j* is the current density (A/cm^2^).

Taking the α-angle into account, the diffusion coefficient of Ni in Sn can be expressed as [9],

(3) $$D_{\mathrm{Ni}(\alpha )}=D_{c}\times \cos^{2}\left(\alpha \right)+D_{a}\times \sin^{2}\left(\alpha \right)$$

(4) $$D_{c}=\left(1.99\pm 0.32\right)\times 10^{-2}\exp\left[-\left(4.32\pm 0.11\;\mathrm{kcal}/\mathrm{mole}\right)/\mathrm{RT}\right]\;{\mathrm{cm}}^{2}/s$$

(5) $$D_{a}=\left(1.87\pm 0.26\right)\times 10^{-2}\exp\left[-\left(12.94\pm 0.12\;\mathrm{kcal}/\mathrm{mole}\right)/\mathrm{RT}\right]\;{\mathrm{cm}}^{2}/s$$

Therefore, we can reformulate as follows,

(6) $$\frac{A\times h}{\Omega_{\mathrm{Ni}}}=J_{\mathrm{EM},\mathrm{Ni}}\times A\times t$$

After expansion, we can obtain,

(7) $$\frac{A\times h}{\Omega_{\mathrm{Ni}}}=C_{\mathrm{Ni}}\times \frac{D_{\mathrm{Ni}(\alpha )}}{kT}\times \left(Z^{*}\times e\times \rho_{\mathrm{Sn}}\times j\right)\times A\times t$$

After removing the shift term, we can get the following equation,

(8) $$\frac{h}{t}=\Omega_{\mathrm{Ni}}\times C_{\mathrm{Ni}}\times \frac{D_{\mathrm{Ni}(\alpha )}}{kT}\times \left(Z^{*}\times e\times \rho_{\mathrm{Sn}}\times j\right)$$

Finally, with the diffusion coefficient of Ni in Sn, we can obtain the theoretical dissolution rate of Ni UBM under the high current density of 8 × 10^4^ A/cm^2^ as,

(9) $$r=\frac{h}{t}=1.78\times 10^{3}\times D_{\mathrm{Ni}(\alpha )}$$

Similarly, the theoretical dissolution rate of Ni UBM with the low current density of 1.6 × 10^4^ A/cm^2^ can be obtained as,

(10) $$r=\frac{h}{t}=3.56\times 10^{2}\times D_{\mathrm{Ni}(\alpha )}$$

According to the above derivation formula, we can calculate the theoretical dissolution rate of Ni. Under the high current density and with an *α*-angle of 0°, the theoretical dissolution rate of Ni UBM is 0.21 (μm/h). When the *α*-angle is 90°, the theoretical dissolution rate is 6.90 × 10^−6^ (μm/h). At the low current density and with an α-angle of 0°, the theoretical dissolution rate of the Ni UBM is 0.04 (μm/h). The theoretical dissolution rate is 1.37 × 10^−6^ (μm/h) with an *α*-angle of 90°. Finally, we can plot the theoretical dissolution rate as a function of *α*-angle, as illustrated in Figure 8b,c.

**Figure 9:**
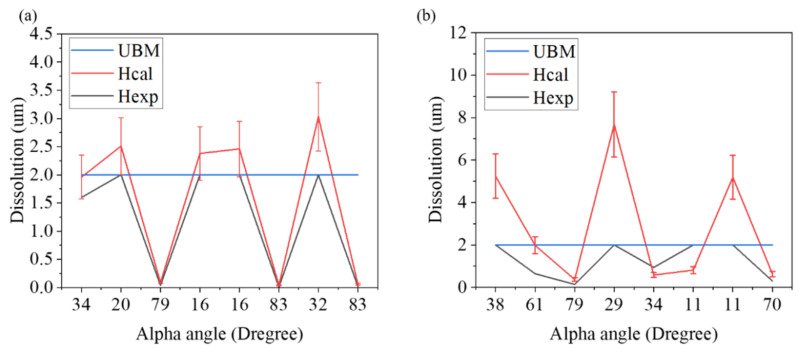
Theoretical versus actual consumption rates for (**a**) 8 × 10^4^ A/cm^2^ and (**b**) 1.6 × 10^4^ A/cm^2^ current density with different *α*-angles of Ni UBM. The thickness of the Ni UBM was only 2 μm. For some of the low angle Sn grains, the theoretical dissolution thickness is over 2 μm, which means that all the 2-μm Ni UBM dissolved after the EM test. The experimental results also supported the theoretic calculation.

We can further correlate the theoretical dissolution rates and α-angles with the experimental data measured by the EBSD images in Figure 6 and Figure 7. The theoretical dissolution rate and the actual consumption rate of Ni UBM under EM stressing at high and low current densities are shown in Table 1 and Table 2, respectively. Obviously, a good agreement between the theoretical modeling and experiments was obtained, as shown in Figure 9. The original thickness of Ni UBM was 2 μm. Thus, we can effectively employ such a theoretical model to evaluate the EM-induced failures of solder joints possessing different grain orientations.

From the comparison of the consumption rate of the nickel cushion layer with high current density, it is found that the theoretical calculation value should consume 0.04 μm of nickel layer at the highest angle measured so far, and the actual calculated value consumes only 0.01 μm, a difference of about 25%. Low angles have the same result. The theoretical value of the lowest angle measured at present should consume 2.46 μm, and the actual calculated value consumes 2 μm, a difference of about 20%.

When comparing the depletion rates of the nickel underlayer at low current densities, although the trend is consistent, the gap between the theoretical value and the actual value becomes larger. The highest angle consumption should theoretically consume 0.37 μm of nickel layer, but in fact, it only consumes 0.14 μm of nickel layer, the difference being about 37%. The lowest angle consumption should theoretically consume 5.19 μm of nickel layer, but in fact, it only consumes 2 μm of nickel layer, the difference being about 38%. Among them, there are outliers in the data of sample 3. It is very likely that the sample itself is defective, which leads to the rapid consumption of tin. It is judged that the phenomenon of “side wetting” may occur.

Electromigration is a phenomenon in which atoms are bombarded by a large number of high-speed electrons. It results in the mass transfer which causes atoms to be pushed from cathode to anode. Under EM stressing, the Ni UBM in solders with a small *α*-angle is unstable and thus weak-resistant to EM-induced failures. The dissolution rates of the Ni UBMs possessing a low α-angle are greater than that of larger *α*-angle ones. Once the Ni UBM is dissolved, Cu can also easily diffuse into the Sn grains to form CuNiSn IMCs. Then, the failure mode is attributed to dissolution of Ni and Cu UBM, as illustrated in Figure 7. On the other hand, when the Ni UBM is adjacent to a Sn grain with a high *α*-angle, the dissolution rate of Ni is low. But the EM wind force may migrate the Sn atoms to the anode end, resulting in void formation in the cathode side. Under a high current density, various voids were commonly observed in the solder joints with low *α*-angles, as shown in Figure 6. Under a low current density, we rarely observed voids. It is speculated that, under low current stressing, the diffusion rate of Ni could catch up with the mass transfer rate of Sn atoms caused by the electron flow. However, at a high current density, the EM rate of Sn atoms is higher in some of the joints, and the Ni dissolution rate is low. Therefore, various voids mostly formed in the cathode end of the solder joints with low α-angles under a high current density.

## 4. Conclusions

In summary, symmetrical solder joints (Cu/Ni/SnAg_2.3_/Ni/Cu) were fabricated and tested at high (8 × 10^4^ A/cm^2^) or low (1.6 × 10^4^ A/cm^2^) current densities. The correlation between grain orientations and EM-induced failures was then addressed. We found that the joints with high *α*-angles were more resistant to EM failures. The diffusion rate of Ni increased with a decrease in the orientation angle *α* of solder grains. A theoretical model was also proposed to calculate the consumption rate of Ni UBM and compared with the experimental results. The difference between the theoretical value and the experimental value of the Ni UBM consumption rate at high current density is about 20%, and the Ni UBM consumption rate at low current density is about 37%. Good agreement between the numerical modeling and experiments was achieved proving the applicability of such a model to evaluate the EM-induced failures of solder joints. Under EM stressing, voids mostly formed at the cathode ends. At a high current density, we observed the formation of voids in the solder joints with low α-angles. However, voids were rarely detected in the joints with low α-angle at low current stressing. 

## Figures and Tables

**Table 1:** Comparison of theoretical and experimental consumption rates of Ni UBM at high current densities.

Sample No.	1		2		3		4	
Kelvin Position	Left	Right	Left	Right	Left	Right	Left	Right
Time (h)	14.23		12.91		13.34		21.10	
Rate (µm/h)	0.137	0.176	0.007	0.185	0.185	0.003	0.144	0.003
*α*-angle	34	20	79	16	16	83	32	83
H (exp., µm)	1.6	2	0.04	2	2	0.01	2	0.02
H (cal., µm)	1.96	2.51	0.09	2.38	2.46	0.04	3.03	0.06

**Table 2:** Comparison of theoretical and actual depletion rates of Ni UBM at low current densities.

Sample No.	1		2		3		4	
Kelvin Position	Left	Right	Left	Right	Left	Right	Left	Right
Time (h)	212.41		252.66		21.16		135.52	
Rate (µm/h)	0.024	0.009	0.001	0.030	0.027	0.038	0.038	0.005
*α*-angle	38	61	79	29	34	11	11	70
H (exp., µm)	2	0.64	0.14	2	0.94	2	2	0.3
H (cal., µm)	5.24	1.98	0.37	7.68	0.58	0.81	5.19	0.63

## Data Availability

Not applicable.
